# Developmental Switch of Leptin Action on Network Driven Activity in the Neonatal Rat Hippocampus

**DOI:** 10.3389/fncel.2019.00254

**Published:** 2019-06-04

**Authors:** Camille Dumon, Lucie Pisella, Diabe Diabira, Yasmine Belaidouni, Gary A. Wayman, Jean-Luc Gaiarsa

**Affiliations:** ^1^INSERM (Institut National de la Santé et de la Recherche Médicale) Unité 1249, UMR 1249, INMED (Institut de Neurobiologie de la Méditerranée), Aix-Marseille University, Marseille, France; ^2^Program in Neuroscience, Department of Integrative Physiology and Neuroscience, Washington State University, Pullman, WA, United States

**Keywords:** GDPs, leptin, CaMK, ERK, BK-channels, PI3K, immature networks

## Abstract

The adipose-derived circulating hormone leptin plays a pivotal role in the control of energy balance and body weight. Sound data indicate that this hormone also acts as an important developmental signal impacting a number of brain regions during fetal and postnatal stages. Leptin levels surge during the two first postnatal weeks of life in rodents. This period is characterized by the presence of early network driven activity in the immature hippocampus, the so-called Giant Depolarizing Potentials (GDPs). GDPs are thought to contribute to the wiring of the hippocampal network. We therefore tested the effect of leptin on GDPs. Leptin increased GDPs frequency between the postnatal days (P) 1 and 3 via a calcium/Calmodulin-dependent kinase (CaMK) and extracellular signal-related kinase (ERK) dependent pathways. Between P6 and P7, leptin inhibited the frequency of GDPs through the activation of large conductance Ca^2+^ activated K^+^ (BK) channels driven by a phosphoinositol-3 kinase (PI3K) dependent pathway. These results show that leptin exerts a bi-directional and age-dependent control of GDPs and extends the scope of leptin’s action in the developing brain.

## Introduction

The adipose hormone leptin regulates feeding and thermogenesis acting on specific hypothalamic nuclei (Ahima and Flier, 2000). The central action of leptin is, however, not restricted to this structure. In the adult hippocampus, leptin hyperpolarizes pyramidal neurons (Shanley et al., 2002a), inhibits epileptiform activity (Shanley et al., 2002b), regulates the strength of glutamatergic (Harvey, 2007) and GABAergic synaptic transmissions (Solovyova et al., 2009; Guimond et al., 2014) and has pro-cognitive (Harvey, 2007) and antidepressant effects (Guo et al., 2012). Leptin levels are developmentally regulated (Ahima and Flier, 2000) and a large body of evidence also indicates that optimal leptin levels are critical during gestation and early postnatal stages in rodent to promote many aspects of neuronal network development (Bouret, 2010; Harvey, 2013; Dumon et al., 2018).

Spontaneous correlated neuronal activity is a hallmark of developing neuronal networks. In the rodent hippocampus, the spontaneous correlated neuronal activity takes the form of Giant Depolarizing Potentials (GDPs), that are present during the two first postnatal weeks of life (Ben-Ari et al., 1989). These events, triggered by the interplay of glutamatergic and GABAergic synapses, are present at late embryonic and early postnatal stages in rodents and have been proposed to be the *in vitro* counter part of the “sharp waves” recorded in rodent pups *in vivo* (Leinekugel et al., 2002). Calcium transients associated with GDPs enhanced the efficacy of developing glutamatergic (Mohajerani et al., 2007) and GABAergic (Kasyanov et al., 2004) synapses and may contribute to the wiring of the hippocampal network (Lauri et al., 2003; Colin-Le Brun et al., 2004; Huupponen et al., 2013).

Leptin levels surge during the early postnatal period in rodents (Ahima and Flier, 2000), concomitantly with the emergence of GDPs (Ben-Ari et al., 1989). The present study was conducted to determine the effects of leptin on GDPs. We show that leptin modulates GDPs frequency and that the effect changed from excitation to inhibition across developmental stages.

## Materials and Methods

### Animals

All animal procedures were carried out in accordance with the European Union Directive of 22 September (2010/63/EU). The protocol was approved by the INSERM Local committee (Number 0287.01, delivered by Ministère de l’Education et de la Recherche). Experiments were performed on male and female postnatal day (P) 1 to 7 Wistar rats, housed in a temperature-controlled environment with a 12 light/dark cycle and free access to food and water.

### Hippocampal Slice Preparation

Brains were removed and immersed into ice-cold (4°C) artificial cerebrospinal fluid (ACSF) with the following composition (in mM): 126 NaCl, 3.5 KCl, 2 CaCl_2_, 1.3 MgCl_2_, 1.2 NaH_2_PO_4_, 25 NaHCO_3_, and 11 glucose, pH 7.4 equilibrated with 95% O_2_ and 5% CO_2_. Hippocampal slices (600 μm thick) were cut with a McIlwain tissue chopper (Campden Instruments, Ltd.) and kept in ACSF at 25°C. Slices were then transferred to a submerged recording chamber perfused with oxygenated (95% O_2_ and 5% CO_2_) ACSF (3 ml/min) at 34°C.

### Electrophysiological Recordings

Extracellular field potentials were recorded using tungsten wire electrodes (diameter: 50 μm, California Fine Wire, Grover Beach, CA, United States) located in the stratum pyramidal of the CA3 region and a low-noise multichannel DAM-8A amplifiers (WPI, GB; low filter: 0.1 Hz; high filter: 3 kHz; × 1000). Whole-cell patch clamp recordings of glutamate mediated post-synaptic currents (EPSCs) were performed from P1–P3 CA3 pyramidal neurons in voltage-clamp mode at a holding potential of -50 mV using an Axopatch 200B (Axon Instrument, United States). The glass recording electrodes (4–7 MΩ) were filled with a solution containing (in mM): 100 KGluconate, 13 KCl, 10 HEPES, 1.1 EGTA, 0.1 CaCl2, 4 MgATP, and 0.3 NaGTP, pH = 7.2 and the osmolality = 280 mOsmol l-1. The signals were digitized using a Digidata1440A convertor (Axon Instruments, United States). pCLAMP1 0.0.1.10 (Axon Instruments, United States) or Axoscope software version 8.1 (Axon Instruments) and MiniAnalysis 6.03 (Synaptosoft, Decatur, CA, United States) programs were used for the acquisition and analysis.

### Statistics

The one way ANOVA followed by a Tukey’s *post hoc* test was used for multiple comparisons between experimental conditions. A two-tailed unpaired Student’s *t*-test was used to analyze difference between two individual groups. A two-tailed paired Student’s *t*-test was used to analyze the effect of leptin on GDPs frequency. Sample size for each group reported in electrophysiological experiments is at least two slices recorded from at least two independent experiments. All data are expressed as Mean ± standard error to the mean (S.E.M.). Data are judged significantly different when *P* < 0.05. In the figures, box plots represent the first and third quartiles; whiskers show data range; horizontal lines show the median and scatter plots show individual data points.

## Results

### Bidirectional Age-Dependent Control of GDPs Frequency by Leptin

**FIGURE 1:**
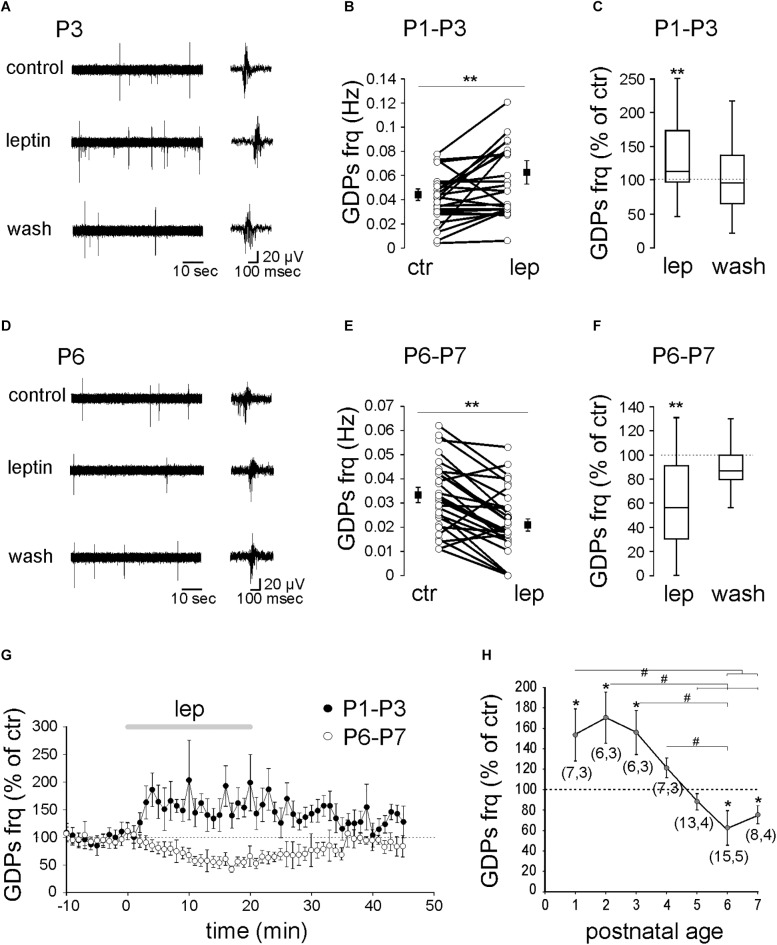
Age-dependent modulation of GDPs frequency by leptin. Extracellular field recordings of GDPs at P3 **(A)** and P6 **(D)** before, during and 20 min after the application of leptin (10 nM). GDPs are shown at an expanded time scale on the right. **(B,E)** Scatter plots showing individual data points obtained between P1–P3 **(B)** and P6–P7 **(E)**. Square filled symbols represent the mean ± SEM. **(C,F)** Box plots of the leptin effect on GDPs frequency between P1–P3 **(C)** and P6–P7 **(F)**. **(G)** Normalized GDPs frequency against time. **(H)** Mean leptin-induced changes of GDPs frequency against age. Numbers in parenthesis indicate the number of slices recorded and animals used. ^∗^*p* < 0.05, ^∗∗^*p* < 0.01 vs. control baseline, paired Student’s *t*-test. ^#^*p* < 0.05, ANOVA followed by a Turkey’s *post hoc* test.

To assess the effect of leptin on GDPs, field recordings were performed on acute rat hippocampal slices from postnatal (P) days 1 to 7. We found that the action of leptin (10 nM) on GDPs changed across postnatal ages. From P1 to P3, leptin increased the frequency of GDPs from 0.044 ± 0.005 to 0.063 ± 0.001 Hz (*n* = 19 slices from nine animals, *P* = 0.01, Figure 1A–C,G). GDPs frequency returned to control level upon the washout of leptin (**Figure 1C,G**). P4–P5 is a transitional period during which leptin had more diverse effect on GDPs frequency (from potentiation to depression) yielding with no significant average effect (0.055 ± 0.004 to 0.053 ± 0.006 Hz, *n* = 20 slices from seven animals, *P* = 0.97, **Figure 1H**). From P6 to P7, leptin depressed the frequency of GDPs from 0.033 ± 0.004 to 0.021 ± 0.002 Hz (*n* = 23 slices from nine animals, *P* = 0.002, **Figure 1D–F**) and returned to control upon the washout (**Figure 1F,G**).

Altogether, these results show that leptin exerts a reversible bidirectional age-dependent control on GDPs frequency (**Figure 1H**).

### Leptin Activates a CaMK- and ERK-Dependent Pathways to Enhance GDPs

**FIGURE 2:**
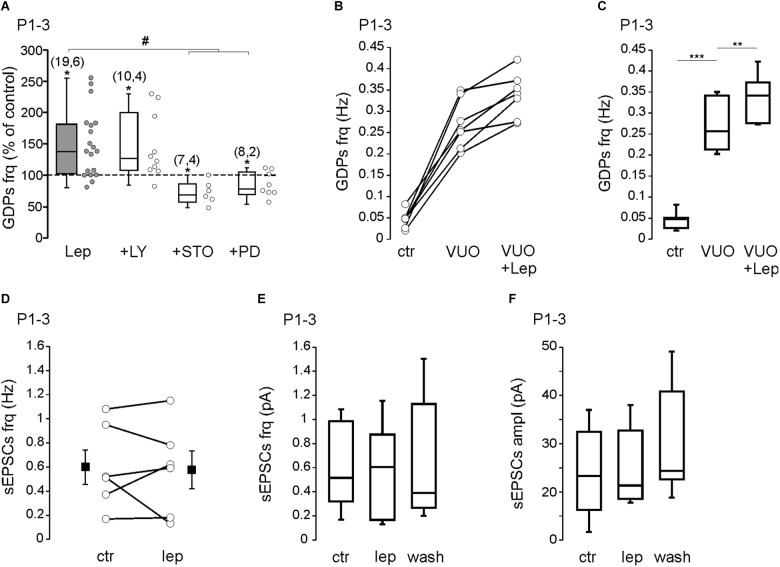
Leptin activates a CaMK- and ERK-dependent pathways to enhance GDPs. **(A)** Box plots of the leptin effect on GDPs frequency at P1–P3 in control condition (Lep, 10 nM) and in the presence of LY294002 (LY, 10 μM), STO-906 (STO, 10 μM), and PD 98059 (PD, 10 μM). **(B,C)** Scatter plots showing individual data points **(B)** and box plots **(C)** of GDPs frequency at P1–P3 in control condition (Ctr), in the presence of VU0463271 (VUO, 10 μM) and VU0463271 + Leptin (VUO+Lep, 10 nM) at P1–P3. **(D)** Scatter plots showing individual data points of the effect of leptin (Lep, 10 nM) on the frequency of sEPSCs at P1–P3. Square filled symbols represent the mean ± SEM. **(E,F)** Box plots of the leptin effect on the frequency **(E)** and amplitude **(F)** of sEPSCs at P1–P3. Numbers in parenthesis indicate the number of slices recorded and animals used. ^∗^*p* < 0.05, ^∗∗^*p* < 0.01, ^∗∗∗^*p* < 0.001 vs. control baseline, paired Student’s *t*-test. ^#^*p* < 0.05 vs. leptin, unpaired Student’s *t*-test.

We next investigated the downstream pathway activated by leptin. Leptin has been reported to activate the extracellular signal-related kinase (ERK), the phosphoinositol-3 kinase (PI3K) and the calcium/CaM-dependent kinase (CaMK) pathway (Harvey, 2007; Solovyova et al., 2009; Dhar et al., 2014; Guimond et al., 2014). We first tested the ability of leptin to enhance GDPs frequency. We found that the potent PI3K inhibitor LY294002 (10 μM) did not prevent the effect of leptin at P1–3 (from 0.055 ± 0.007 to 0.075 ± 0.007 Hz, *n* = 10 slices from four animals, *P* = 0.02, **Figure 2A**). Blocking CaMKK activity with STO-609 (10 μM) or MEK activity with PD 98059 (10 μM), however, abolished the potentiating effect of leptin and uncovered a depression of GDPs [from 0.08 ± 0.02 to 0.06 ± 0.01 Hz in the presence of STO-609 (*n* = 7 slices from four animals, *P* = 0.01) and from 0.082 ± 0.007 to 0.062 ± 0.006 Hz in the presence of PD 98059 (*n* = 8 slices from two animals, *P* = 0.04, **Figure 2A**)]. None of the kinase inhibitors, but STO-609, had a significant effect on the basal frequency of GDPs at P1–P3 (Supplementary Table 1).

In previous studies we reported that leptin potentiates GABAergic synaptic transmission (Guimond et al., 2014) and down-regulates the activity of the K^+^–Cl^-^ cotransporter KCC2, promoting the depolarizing action of GABA (Dumon et al., 2018) in the developing rodent hippocampus. Moreover, leptin has been reported to modulate the strength of hippocampal glutamatergic synapses (Moult and Harvey, 2011; Luo et al., 2015). Because GDPs are triggered by the interplay of glutamatergic and GABAergic synapses, modifying their strength may have important effect on GDPs frequency. The potentiation of GABAergic activity by leptin is abolished by the PI3K inhibitor LY294002 (Guimond et al., 2014) and thus is unlikely to account for the PI3K-resistant increase of GDPs frequency (**Figure 2A**). We next sought to determine whether a down-regulation of KCC2 activity could play a role in the leptin-induced increase of GDPs frequency. As already reported (Spoljaric et al., 2019), we found that the KCC2 blocker VU0463271 (10 μM) induced a significant increase of GDPs frequency (from 0.046 ± 0.007 to 0.27 ± 0.02 Hz, *n* = 7 slices from three animals, *P* = 0.00007, **Figure 2B,C**) but it did not prevent the effect of leptin (from 0.27 ± 0.02 to 0.33 ± 0.02 Hz, *P* = 0.002, **Figure 2B,C**). We also assessed whether leptin could affect glutamatergic synapses. We found that bath applied leptine (10 nM, 20 min) had no significant effect on the frequency (from 0.59 ± 0.14 to 0.57 ± 0.15 Hz) and amplitude (from 24.2 ± 3.2 to 24.8 ± 3.2 pA) of spontaneous glutamatergic activity recorded on P1–P3 CA3 pyramidal neurons (*n* = 6 from two animals, *P* = 0.7 for both, **Figure 2D–F**).

Altogether, these results show that leptin increases the frequency of GDPs through the activation of a CaMK- and ERK-dependent pathway at P1–3, that does not involved the GABAergic and glutamatergic synaptic activity.

### Leptin Inhibits GDPs Through a PI3K-Dependent Activation of Large Conductance Ca^2+^-Activated K^+^ (BK) Channels

**FIGURE 3:**
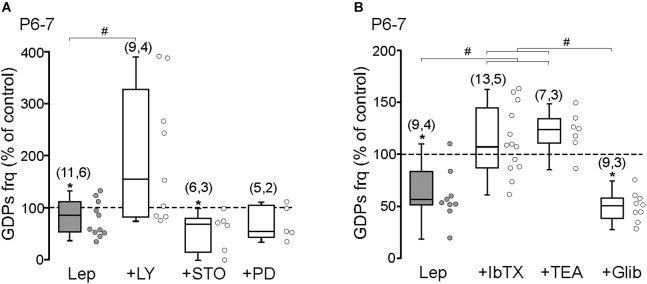
Leptin inhibits GDPs through a PI3K-dependent activation of large conductance Ca^2+^-activated K^+^ (BK) channels. **(A)** Box plots of the leptin effect on GDPs frequency at P6–P7 in control condition (Lep, 10 nM) and in the presence of LY294002 (LY, 10 μM), STO-906 (STO, 10 μM), and PD 98059 (PD, 10 μM). **(B)** Box plots of the leptin effect on GDPs frequency at P6–P7 in control condition (Lep, 10 nM) and in the presence of Iberiotoxin (IbTx, 2 nM), TEA (1 mM), and Glibenclamide (Glib, 10 μM). Numbers in parenthesis indicate the number of slices recorded and animals used. ^∗^*p* < 0.05 vs. control baseline, paired Student’s *t*-test. ^#^*p* < 0.05 vs. leptin, unpaired Student’s *t*-test.

We next tested the ability of leptin to inhibit the frequency of GDPs at P6–7. The results show that the PI3K inhibitor LY294002 (10 μM) abolished the leptin-induced depression of GDPs (0.051 ± 0.02 to 0.061 ± 0.02 Hz, *n* = 9 slices from four animals, *P* = 0.32, **Figure 3A**). Neither the CaMK inhibitor STO-609 (10 μM) nor the MEK inhibitor PD 98059 (10 μM) prevented the leptin-induced depression of GDPs: from 0.048 ± 0.015 to 0.029 ± 0.013 Hz in STO-609 (*n* = 6 slices from three animals, *P* = 0.048) and from 0.032 ± 0.006 to 0.021 ± 0.004 Hz in PD 98059 (*n* = 5 slices from two animals, *P* = 0.044, **Figure 3A**). None of the kinase inhibitors, but STO-609, had a significant effect on the basal frequency of GDPs at P6–P7 (Supplementary Table 1).

Adult hippocampal pyramidal neurons and hypothalamic NPY/AgRP neurons are inhibited by leptin through respectively the activation of large conductance Ca^2+^-activated K^+^ (BK) channels (Shanley et al., 2002a) and ATP-sensitive K^+^ (K_ATP_) channels (Spanswick et al., 1997) via PI3K. We therefore examined the effect of Iberiotoxin (IbTx, 2 nM) a potent BK channels antagonist on the leptin-induced depression of GDPs at P6–7. IbTx abolished the effect of leptin on GDPs frequency (0.048 ± 0.008 to 0.051 ± 0.004 Hz, *n* = 13 slices from five animals, *P* = 0.47, **Figure 3B**). The same result was observed with the potassium channel blocker TEA (1 mM, from 0.057 ± 0.021 to 0.071 ± 0.03 Hz, *n* = 7 slices from three animals, *P* = 0.42, **Figure 3B**). However, the K_ATP_ channels antagonist Glibenclamide (10 μM) did not prevent the action of leptin (from 0.055 ± 0.012 to 0.029 ± 0.006 Hz, *n* = 9 slices from three animals, *P* = 0.004, **Figure 3B**). None of the K^+^ channel blockers had a significant effect of the basal frequency of GDPs at P6–P7 (Supplementary Table 1).

These results show that leptin inhibits the frequency of GDPs through a PI3K-dependent activation of BK channels at P6–7.

## Discussion

In the present study, we examined the ontogeny of leptin’s action in the developing rat hippocampus. We found that leptin exerts a bidirectional age-dependent modulation of GDPs during the first postnatal week of life. Although we cannot exclude unspecific effects of leptin on other cytokine receptors, a direct effect of leptin on specific receptors is supported by the facts that the full-length isoform leptin receptor B (LepRB, the only leptin receptor able to activate intracellular pathway) is present and functional in the developing hippocampus (Shanley et al., 2002b; Guimond et al., 2014) and that leptin modulates GDPs frequency at a low concentration (10 nM).

The mechanism underlying the ability of leptin to inhibit GDPs at P6–7 involves stimulation of PI3K and activation of BK channels since the inhibitory action of leptin was blocked by the PI3K inhibitor LY294002 and the BK blocker Iberiotoxin. K_ATP_ channels are important molecular targets of leptin receptors in the hypothalamus (Spanswick et al., 1997). However, the K_ATP_ channel blocker did not prevent the leptin-induced inhibition of GDPs. Previous studies reported that leptin inhibits the firing of adult hippocampal neurons through a PI3K-dependent reorganization of actin filaments and subsequent membrane clustering of BK channels (Shanley et al., 2002a; O’Malley et al., 2005). This signaling pathway provides a mechanism by which leptin inhibits epileptiform-like activities in the adult hippocampus (Shanley et al., 2002b) and may contribute to the leptin-induced inhibition of GDPs in the developing hippocampus.

We found that the activation of CaMK and MEK-dependent pathways are required for leptin to enhance GDPs at P1–P3, but the identification of the signaling partners involved awaits further explorations. Leptin enhances the strength of GABAergic (Solovyova et al., 2009; Guimond et al., 2014) and glutamatergic (Moult and Harvey, 2011; Luo et al., 2015) synapses and promotes a depolarizing action of GABA (Dumon et al., 2018). However, these mechanisms are unlikely to contribute to the leptin-induced increase of GDPs frequency since (1) the potentiation of GABAergic and glutamatergic activity, but not the leptin-induced enhancement of GDPs, were abolished by the PI3K inhibitor LY294002 (Solovyova et al., 2009; Moult and Harvey, 2011; Guimond et al., 2014; Luo et al., 2015), (2) the depolarizing shift of GABA responses induced by leptin was abolished by the KCC2 blocker VU0463271 (Dumon et al., 2018), while VU0463271 didn’t prevent the leptin-induced increase in GDPs frequency and (3) we found that leptin had no significant effect of the glutamatergic activity recorded on P1–P3 CA3 pyramidal neurons. Leptin has been reported to depolarize cultured hippocampal neurons through the activation of TrpC channels driven by a CaMK-dependent pathway (Dhar et al., 2014). Activation of TrpC channels may underlie the leptin-induced enhancement of GDPs frequency. SKF96365, a general non selective TrpC channel blocker, completely abolished the GDPs (unpublished results), preventing us from testing this hypothesis.

It is worth noting that while leptin increases the frequency of GDPs at P1–P3 in control conditions the same application in the presence of STO-609 or PD 98059 uncovers an inhibitory action (**Figure 2A**). Likewise, at P6-P7, leptin inhibits GDPs in control conditions, but enhances their frequency in the presence of LY294002, Iberiotoxin or TEA (**Figure 3**). These results suggest that both the enhancing and inhibiting actions of leptin on GDPs frequency co-exist during the postnatal period. The cellular/molecular basis for the switch of leptin action is unclear. One possible explanation could be that leptin activates distinct leptin receptors, differing in their location (i.e., pre- vs. post-synaptic or pyramidal cells vs. interneurons), signaling molecules and/or downstream effectors, which exhibit different developmental profiles of expression or activity. Accordingly, developmental switches of leptin actions on glutamatergic synaptic transmission have been reported previously through the activation of different downstream pathways and effectors (Moult and Harvey, 2011; Luo et al., 2015). Likewise, leptin causes a membrane depolarization of NPY/AgRP/GABA neurons in the hypothalamic arcuate nucleus in juvenile mice, rather than the expected hyperpolarization observed in adults (Baquero et al., 2014).

The function of leptin control over the frequency of GDPs is currently unknown, but may contribute to the wiring of the hippocampal network. In previous studies we reported that leptin plays a crucial role in the emergence of functional GABAergic inhibition in the rodent hippocampus (Guimond et al., 2014; Dumon et al., 2018). The present study further extends the scope of leptin’s action in the developing hippocampus and brings new perspectives for its potential implication in brain development.

## Data Availability

All datasets generated for this study are included in the manuscript and/or the Supplementary Files.

## Author Contributions

J-LG, GW, LP, and CD conceived and designed the experiments, and wrote the manuscript. LP, CD, YB, and DD performed the experiments, and analyzed the data. All authors approved the final version of the manuscript.

## Conflict of Interest Statement

The authors declare that the research was conducted in the absence of any commercial or financial relationships that could be construed as a potential conflict of interest.
