# Supplementary material for: Developmental Switch of Leptin Action on Network Driven Activity in the Neonatal Rat Hippocampus
Source: Front Cell Neurosci. 2019 Jun 4;13:254. doi: 10.3389/fncel.2019.00254 (PMC6558146; doi:10.3389/fncel.2019.00254)
Supplement: Supplementary file 1 [file Table_1.DOCX]

**Supplementary table 1**: Effect of the kinase inhibitors and potassium channel blockers on the basal frequency of GDPs. Data are expressed as Mean + standard error to the mean (S.E.M.). A two-tailed paired Student’s *t*-test was used to analyze the effect on GDPs frequency. N= number of slices tested. n. d.; not done.

|  | P1-3 | P6-7 |
| --- | --- | --- |
| STO-609 (10 µM) | 0.053+0.04 to 0.089+0.01 Hz (P=0.046, n=7) | 0.03+0.01 to 0.048+0.015 Hz  (P=0.048, n=6) |
| LY294002 (10 µM) | 0.06+0.012 to 0.059+0.007 Hz  (P=0.8, n=5) | 0.051+0.02 to 0.052+0.02 Hz  (P=0.79, n=9) |
| PD 98059 (10 µM) | 0.076+0.009 to 0.082+0.007 Hz  (P=0.25, n=8) | 0.028+0.004 to 0.032+0.006 Hz  (P=0.09, n=5) |
|  |  |  |
| Iberiotoxin (2µM) | n.d. | 0.044+0.01 to 0.048+0.008 Hz  (P=0.047, n=12) |
| TEA (1 mM) | n.d. | 0.039+0.005 to 0.057+0.021 Hz  (P=0.42, n7) |
| Glibenclamide (10 µM) | n.d. | 0.047+0.005 to 0.055+0.012 Hz  (P=0.27, n=9) |
